# Timely Completion of Inpatient Discharge Summaries: A Clinical Audit

**DOI:** 10.1192/bjo.2026.11675

**Published:** 2026-06-30

**Authors:** Kerion Komolafe

**Affiliations:** South West Yorkshire Foundation Trust, Barnsley, United Kingdom

## Abstract

**Aims:**

To evaluate compliance with the Trust standard for completion of inpatient discharge summaries within 24 hours of discharge and to identify reasons for delay.

**Methods:**

A retrospective clinical audit was conducted across two adult inpatient psychiatric wards over a four-month period (28 May–26 September). Fifty consecutive inpatient discharges were sampled (Clark Ward n=25; Beamshaw Ward n=25). Discharge date/time was compared with the time of completion of the EPMA full discharge summary. Outcomes assessed included timeliness of completion, diagnostic accuracy, and reasons for delay.

**Results:**

Overall, 76% (n=38) of discharge summaries were completed within 24 hours, with 24% (n=12) delayed, falling below the Trust compliance threshold of >91%. The most common reason for delay was an unclear EPMA discharge process, where summaries were signed off but not fully finalised (n=5). Other contributing factors included staffing shortages (n=1), awaiting investigation results (e.g. CT head report; n=1), transfer to community services under a Community Treatment Order (n=1), absence of a junior doctor at the discharge meeting (n=1), and unspecified causes (n=3).

**Conclusion:**

While the majority of inpatient discharge summaries were completed within the required timeframe, overall compliance did not meet Trust standards. System-related barriers–particularly ambiguity within the EPMA workflow–were the predominant contributors to delay. Targeted interventions, including reinforcing EPMA discharge processes, regular MDT-linked updates, proactive review of anticipated discharges, and visible guidance for clinicians, are recommended. These measures aim to improve efficiency, reduce avoidable delays, and achieve sustained compliance above 91%, enhancing patient safety and continuity of psychiatric care.

